# The feasibility of direct adductor canal block (DACB) as a part of periarticular injection in total knee arthroplasty

**DOI:** 10.1186/s43019-020-00066-z

**Published:** 2020-09-21

**Authors:** Vaibhav Bagaria, Rajiv V. Kulkarni, Anisha Valavi, Himanshu Choudhury, Anoop Dhamangaonkar, Dipit Sahu

**Affiliations:** 1 Department of Orthopaedics, Sir H N Reliance Foundation Hospital, Mumbai, India; 2 Department of Radiology, Sir H N Reliance Foundation Hospital, Girgaum, Mumbai, India; 3 Department of Orthopaedic Surgery, LTMG Hospital, Sion, Mumbai, India

**Keywords:** Total knee arthroplasty, Periarticular injection, Adductor canal block, Ultrasound, Pain management

## Abstract

**Background:**

Adductor canal block (ACB) is one of the preferred methods of analgesia in total knee arthroplasty (TKA). However, conventionally its use is time-consuming, requires ultrasound guidance, a trained anaesthesia team and adherence to strict asepsis by members of the allied teams. This study was done to assess the feasibility and safety of direct adductor canal block (DACB) as a part of surgeon-administered periarticular infiltration.

**Materials and methods:**

Thirty computed tomography (CT) angiography films of the patients were retrospectively reviewed. The trajectory of the needle placement for a DACB in relation to the target region of the adductor block was determined. Fourteen knees in seven cadavers, were dissected through a medial parapatellar approach to perform TKA. After administering the DACB using the technique based on CT data, dissection was carried out to ascertain the correct placement of the dye by visualising the stained areas.

**Results:**

The angle of approach in the coronal plane from the entry point to the medial high point and to the adductor hiatus was 10.2° (8−14°) and 6° (3.8−11°), respectively. The angle of approach in the sagittal plane from the entry point to the medial high point and to the adductor hiatus was 7° (5−10.5°) and 29° (19−43°), respectively. In all the 14 cadaveric knees, we confirmed the correct placement of the methylene blue dye as demonstrated by the staining of the adductor canal.

**Conclusion:**

The study demonstrates the feasibility of the DACB. This surgeon-driven technique is likely to reduce the cost of the procedure, reduce operating room time and also eliminate the risks of surgical-site contamination.

## Introduction

Early recovery after total knee arthroplasty (TKA) is judged by the ability of the patient to walk without pain after the surgery [1]. Early ambulation after TKA is desirable to avoid complications and achieve faster recovery of the range of motion of the knee [2]. However, postoperative pain after TKA is often moderate to severe and limits this goal [3]. Several modalities have been utilised to decrease postoperative pain such as epidural analgesia, systemic analgesia, periarticular injection (PAI), femoral nerve block (FNB) and adductor canal block (ACB) [3–6]. Whereas, the systemic analgesia through opioids can have systemic side effects like vomiting and sedation [7], the FNB can cause quadriceps weakness [8] and occasional falls [9]. Multimodal analgesia including PAI, on the other hand, is a surgeon-administered, quick, effective and inexpensive method providing significant pain relief in the postoperative period [10]. PAI has evolved over years and today utilises a cocktail of drugs such as local anaesthetic agents, opioids and epinephrine, and selectively blocks the sensory nerves [10]. PAI is often combined with other modalities to extend the duration of pain relief. Anaesthesiologist-administered ACB under ultrasound guidance (USG) is also one of the commonest additional modalities that has been effectively utilised to provide analgesia, while maintaining quadriceps strength [11]. ACB given at the targeted region under USG selectively blocks the saphenous nerve in the adductor canal while sparing the motor branches of the femoral nerve [12]. The adductor canal is located in the middle third of the thigh and contains branches of the femoral nerve, the saphenous nerve, the nerve to the vastus medialis, and the articular contribution of the obturator nerve [12]. Since the ACB under USG is administered by an anaesthesiologist, the associated factors, such as time, economy and skill, may potentially limit its widespread use by the surgeons. Recently, Pepper et al. have studied the feasibility of a surgeon-administered direct adductor canal block (DACB) in cadaveric knees [13]. It is postulated that a DACB can be effectively and safely performed, without any additional USG and is based on the anatomic location of the adductor canal.

The aim of our study was to understand the surgical anatomy of the target region of the adductor canal in relation to the periarticular injection trajectory using computed tomography (CT) scan image data. Further, this inference was applied to study the feasibility of a direct ACB as a part of PAI during TKA performed in cadaveric specimens.

## Materials and methods

The study was done in two parts: (1) a computerized tomography angiography (CTA)-based study and (2) a cadaveric study.

### Computerised tomography angiography (CTA) study

A retrospective analysis of 30 CTA scans of the thigh in 30 patients (16 male:14 female) with an average age of 62 years (range 35–90 years), average height of 159.9 cm (143–174 cm) and average weight of 69.8 kg (54 to 100 kg) was done to study the direction and approach to the adductor canal through the supratrochlear area of the knee joint. Three-dimensional (3D) CTA [1] scan images of the thigh were studied to decide the angle of approach to the adductor canal from just above the patella in the region of the medial thigh.

The CTA scan images were entered in the Aquarius iNtuition version .4.4.11.338.8687 software for all measurement studies. The images were reformatted in the coronal, sagittal and axial planes.

**Fig. 1:**
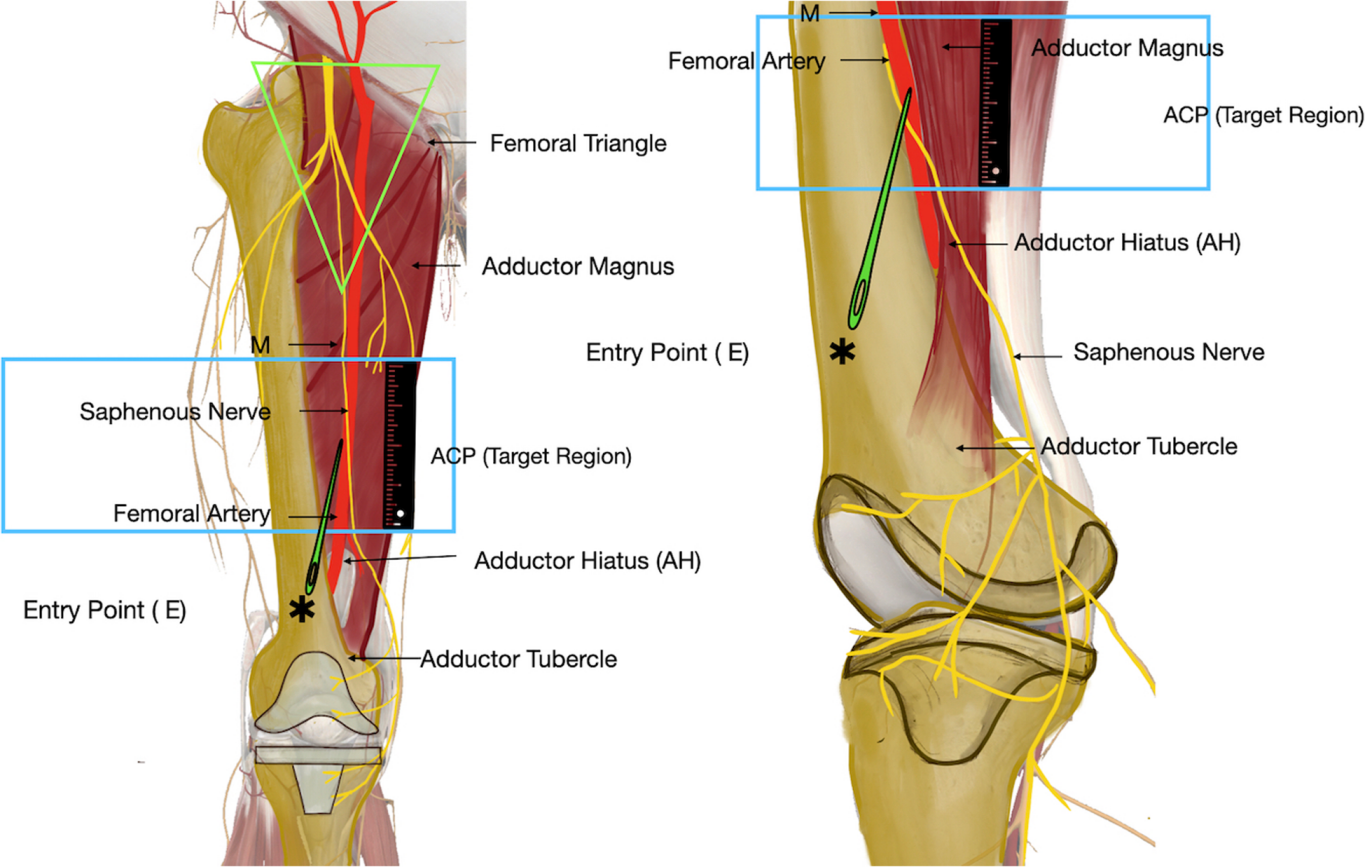
Landmarks and parameters used for measurement. ACP − adductor canal proper: this area extends between the point where the femoral artery changed its course from anterior to medial (M) and exited the canal at the adductor hiatus. Adductor hiatus: this point was marked as the point where the adductor magnus inserts into the femur and just disappears in the axial section. E: the entry point of the needle is 8 cm above the joint line along the antero-medial border of the femur

Since the femoral artery runs through the adductor canal, it was used as a surrogate marker for the trajectory of the adductor canal in the CTA images. The following reference points were established (Fig. 1):

1. Target region or adductor canal proper (ACP): the area of interest for administering the ACB has been called the adductor canal proper (ACP) by Bendtsen et al. [14].
2. Medial high point of the ACP (M): the highest point of the ACP was marked on the medial thigh where the femoral artery changes its course and comes to lie exactly medial to the centre of the femoral diaphysis in the axial section.
3. Adductor hiatus (AH) or low point of the ACP: the point where the femoral artery exits the medial compartment and becomes posterior to the femur
4. Entry point of needle (E): from the surgeon’s experience it was decided that a point that is 8 cm above the joint along the anteromedial border of the femur would be considered the entry point for the DACB.

**Fig. 2:**
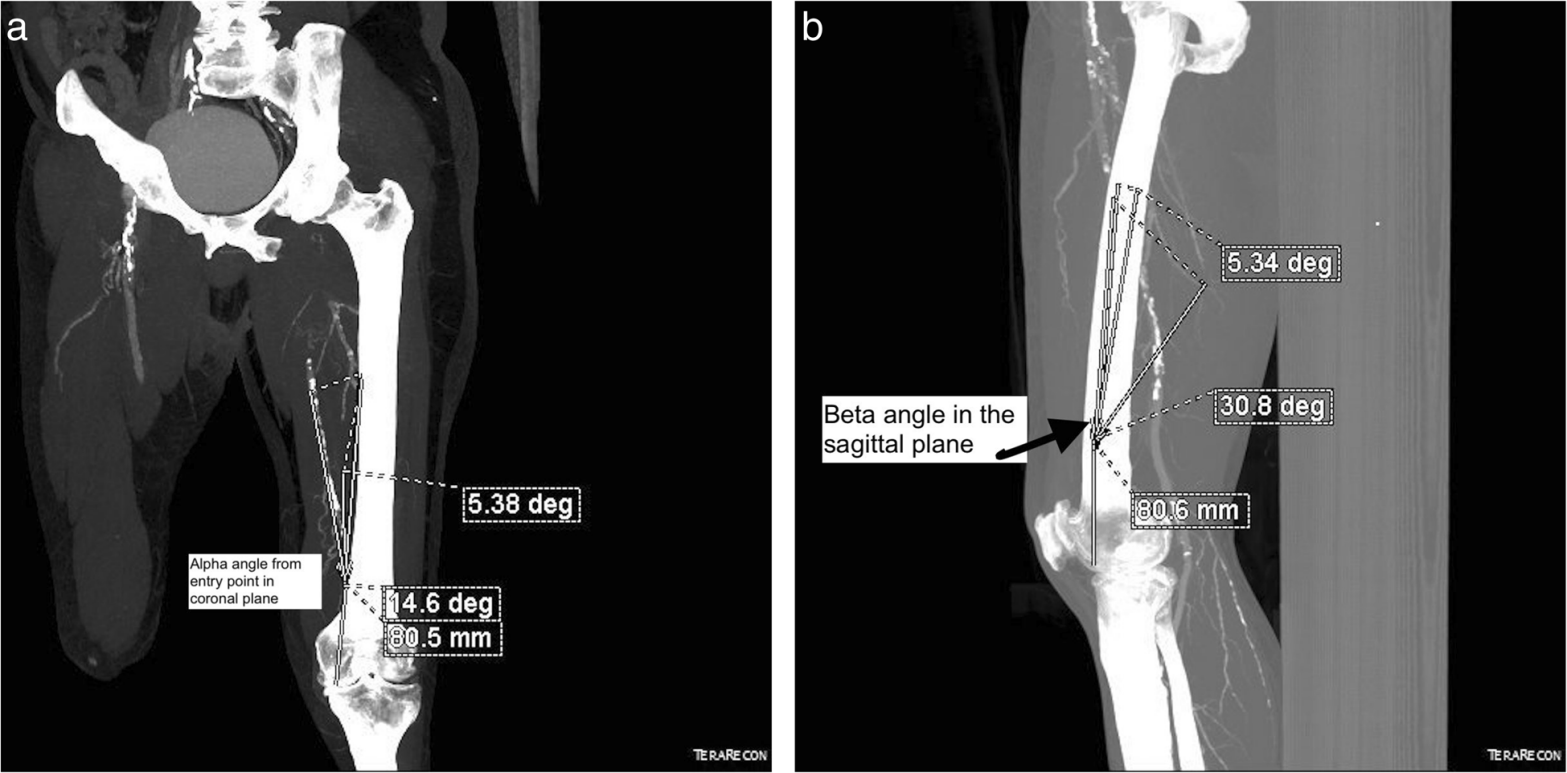
**a** Measurements of the alpha angle taken in computed tomography (CT)-angiogram reformatted images. In the coronal plane a line was drawn from the entry point (E) to the high medial point (M). The angle between this line and the line along the long axis of femur was termed the alpha 1 angle. Another line was drawn in the coronal section from the entry point (E) to the adductor hiatus (AH). The angle between this line and the line along the long axis of the femur was termed the alpha 2 angle. **b** Measurements of the beta angle taken in CT-angiogram reformatted images. Beta angle: in the sagittal plane a line was drawn from the E to the M. The angle between this line and the line along the long axis of the femur was termed the beta 1 angle. Another line was drawn from E to AH. The angle between this line and the long axis of the femur was termed the beta 2 angle

The following parameters were measured:

1. The length of the line joining the entry point (E) to the medial high point (M)
2. The length of the line joining the entry point (E) to the adductor hiatus (AH)
3. The following angles were measured (Fig. 2a, b):
  - Alpha angle: in the coronal plane a line was drawn from the E to the M. The angle between this line and the line along the long axis of the femur was termed the alpha 1 angle. Another line was drawn in the coronal section from the E to the AH. The angle between this line and the line along the long axis of the femur was termed the alpha 2.
  - Beta angle: in the sagittal plane a line was drawn from the E to the M. The angle between this line and the line along the long axis of the femur was termed the beta 1 angle. Another line was drawn from the E to the AH. The angle between this line and the long axis of the femur was termed the beta 2 angle.

### Cadaveric study

**Fig. 3:**
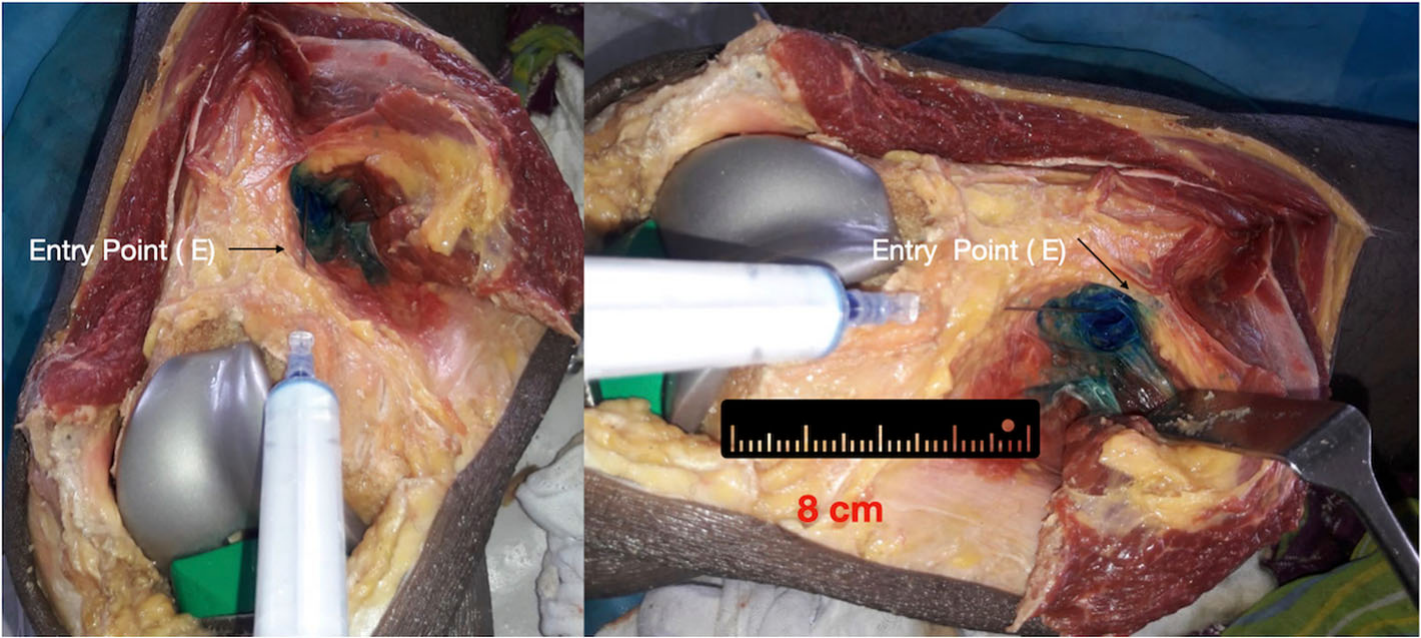
The direct adductor canal block (DACB) injection technique in cadavers. The entry point of the needle is 8 cm above the joint line along the antero-medial border of the femur. It corresponds to 2 finger breadths above the anterior flange of the prosthesis

Seven paired cadaveric knees were dissected as a part of a continuing medical education (CME) programme on arthroplasty. The cadavers were free from any pathology on gross observation. The knees were exposed through a midline parapatellar approach. The patella was retracted laterally and the knee was flexed to expose the distal femur and proximal tibia. The femoral and tibial cuts were performed as per the standard guidelines for TKA. The trial femoral and tibial prostheses were inserted without cement. The entry point for the injection was marked 8 cm above the distal end of the femoral trial. This usually corresponds to two finger breadths above the proximal border of the femoral component. A syringe filled with 5 ml methylene blue and a number-22 spinal needle was used for the DACB. The needle was directed 6−10° medially and 10−30° posteriorly in the proximal direction from the designated entry point. Initially, a goniometer was used to get an idea of the angle of trajectory in the pilot experiment and then all the subsequent placements were based on the surgeon’s judgement. The goniometer was advanced 6–7 cm in the targeted direction (Fig. 3). Then, after aspiration to confirm the absence of blood in the syringe, 5 ml of the methylene blue was injected. While injecting, the needle was withdrawn simultaneously to cover the entire injection track. In order to ascertain the area stained with this injection and to confirm that it corresponded with the targeted area of interest, a medial dissection was carried out. The midline knee incision was extended proximally and medially as far as the middle of the thigh. The skin and subcutaneous tissue were dissected to expose the sartorius muscle. The muscle was carefully retracted to expose the neurovascular bundle underneath. The saphenous nerve, femoral artery and femoral vein were identified. The anatomic structures that were discoloured blue in colour were noted. The presence of dye in the target region that corresponded to the lower half of the adductor canal was recorded as present or absent; partial or complete. Complete staining was defined as the presence of more than 5 cm of staining of the lower half of the adductor canal. After this, the femoral artery and vein were incised to note the presence of any intra-arterial or intravenous injection/staining.

### Data analysis

All continuous data were recorded as mean and range of distribution. All the data were rounded off to one decimal point of precision. Student’s *t* test was used to detect any significant difference between the male and the female population. A *p* value < 0.05 was considered significant.

## Results

### Computerised tomography angiography (CTA) study (Table 1)

The length of the line joining the reference entry point (E) to the medial high point (M) was 15.1 cm. (10.4–23.0 cm) and the length of the line joining the entry point to the adductor hiatus (AH) was 6.9 cm (4–12.4 cm).

**Table 1:** Table shows the results of the computerized tomography angiography (CTA) measurements

Parameter measured	Mean	Range
Entry point to medial high point (in centimetres)	15.1	(10.4–23.0)
Entry point to adductor hiatus (in centimetres)	6.9	(4–12.4).
Alpha 1 − angle in coronal plane (entry point to medial high point) (in degrees)	38°	(28−47°)
Alpha 2 − angle in coronal plane (entry point to adductor hiatus) (in degrees)	24°	(13.6−33.4°)
Beta 1 − angle in sagittal plane (entry point to medial high point) (in degrees)	7°	(5−10.5°)
Beta 2 − angle in sagittal plane (entry point to adductor hiatus) (in degrees)	29°	(19−43°)

Student’s *t* test revealed no significant difference between male and female participants in any of the above two measurements (*p* = 0.7, *p* = 0.3, respectively).

The angle of approach in the coronal plane from the entry point to the medial high point (alpha 1) and to the adductor hiatus (alpha 2) was 10.2° (8−14°) and 6° (3.8−11°), respectively.

The angle of approach in the sagittal plane from the entry point to the medial high point (beta 1) and to the adductor hiatus (beta 2) was 7° (5−10.5°) and 29° (19−43°), respectively.

Student’s *t* test revealed no significant difference between male and female participants in the above-mentioned measurements (*p* = 0.5 for alpha 1, *p* = 0.6 for alpha 2, *p* = 0.6 for beta 1, *p* = 0.8 for beta 2).

### Cadaveric study

**Fig. 4:**
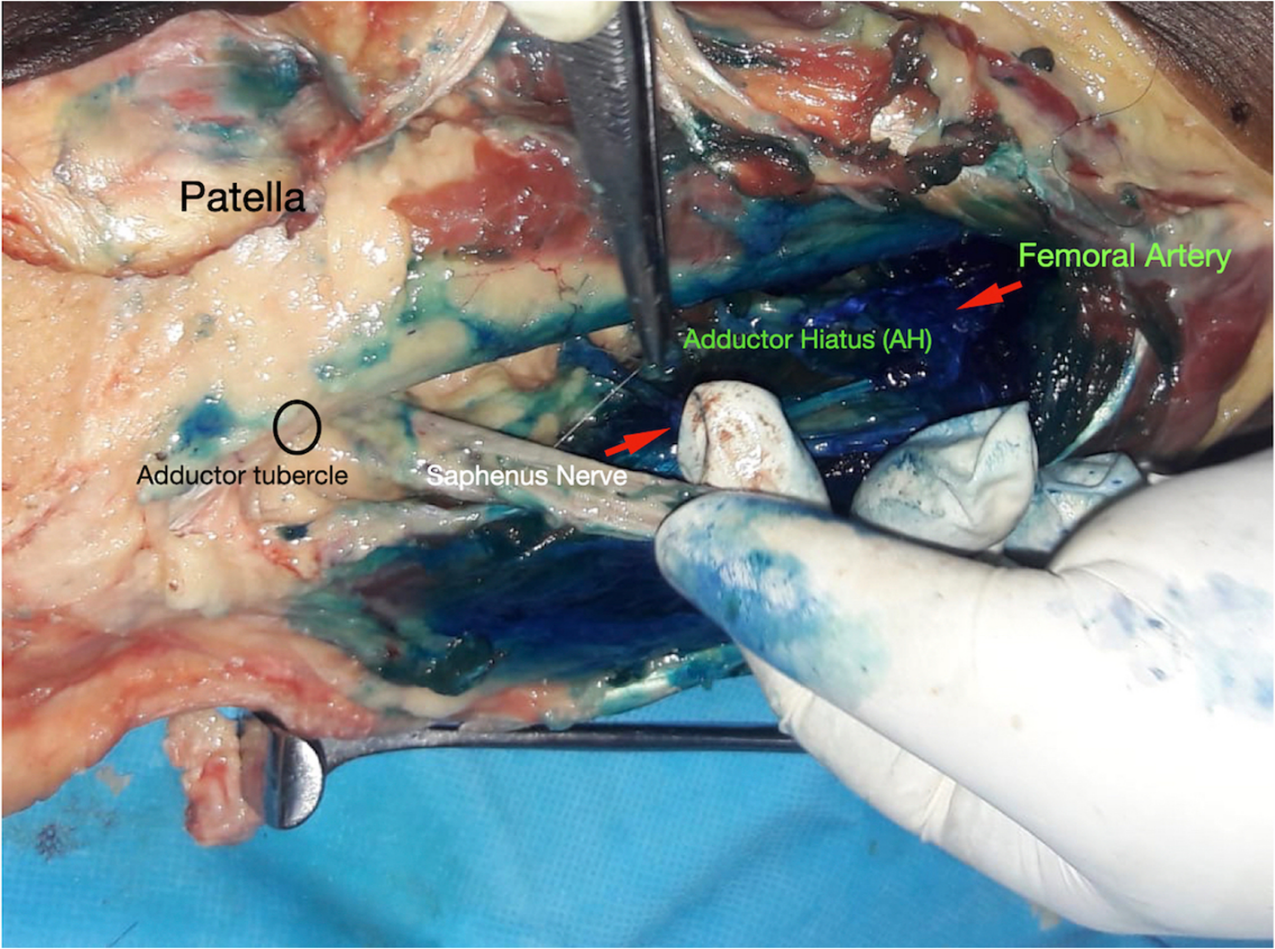
A medial dissection done post injection reveals staining of all the vital structures including the saphenous nerve. No intravascular placement was noted in any cadaver

In all 14 knees (seven cadavers) the methylene blue injection resulted in staining of the lower half of the adductor canal – the target region (Fig. 4). There was complete staining (> 5-cm staining) of the canal in all the knees. All the structures of the lower half of the canal − the femoral artery, the femoral vein and the saphenous vein − could be identified in all the cadavers. In two knees, the nerve to vastus medialis could be identified as stained. Intra-arterial and intravenous dissection of the femoral vessels did not reveal the presence of methylene blue in any of the cases, implying that there was no intravascular injection of the dye.

## Discussion

Surgeon-administered PAI today remains one of the preferred methods of regional anaesthesia for postoperative pain control [10, 15, 16]. It is inexpensive, does not significantly increase the operative time and has a low risk of extraneous contamination. Our study introduces a new route of ACB which can be done as a part of the periarticular infiltration by the operating surgeon. We have shown that this technique of administering a DACB through surgical incision is feasible and the canal is accessible from the same operative wound of TKA surgery. This intraoperative block can be achieved by targeting the adductor canal from the supratrochlear region with the help of a spinal needle. Our CTA study showed that the target area for the block lies at an angle of 7−29° in the sagittal plane and 6−10° in the coronal plane. Our cadaveric study confirmed its feasibility and reproducibility in achieving consistent success in reaching the target region after following the described technique. Furthermore, we also showed that injecting via this approach leads to an appropriate infiltration (evidenced by observable blue discolouration in the cadaver) around the nerves in the canal. The safety of this technique was established as no vessels were breached during our procedure in the cadavers. We demonstrated complete spread of the injection dye around the saphenous nerve in the medial thigh. This is the logical target of the nerve block which has been stated in earlier studies to spare the motor nerves, while effectively blocking the pain sensation in the knee after TKA [14]. The perfect site for ACB has been debated by several authors. Some studies have shown that a proximal ACB block also leads to effective pain block [17]. However, the motor nerves leave the adductor canal only at the mid-thigh, hence a block distal to this location will effectively spare the motor nerves. The mid-thigh and distal third of the thigh has been the region for the administration of ACB by several the authors [9, 18]. Our technique of ACB can be combined with the regular PAI injection that is administered during TKA. This will not only save operating room time, but also save the costs of an additional USG procedure, spare the efforts of extra anaesthesia personnel; thus, making it more widely acceptable. While the use of USG to reach the target region is a proven technique in regional anaesthesia manuals, the case of ACB is unique. In this we are targeting a large cylindrical area that spans a mean length of 15 cm [13] that may not need precise USG-guided localisation. It is also pertinent to note that this block is in effect an infiltration that is supposed to desensitise up to 10 sensory nerves that are present in the target region [18].

Though the ACB results in less quadriceps weakness than FNB, it needs to be administered under ultrasound by an anaesthesiologist [19]. But, Biswas et al. and Nader et al. showed that ACB had better pain scores and lower opioid consumption than PAI alone [20, 21]. Goytizolo et al. also showed that combining PAI and ACB resulted in better pain scores [22]. Hence, there seems to be a clear benefit in combining PAI and ACB through the same intraoperative route to economise on costs and save time; thus, making it more efficient. A similar study on cadaveric knees by Pepper et al. also showed that an intraoperative DACB block at the distal location is feasible through the knee joint [13]. However, they studied the procedure on native knee joints without prosthetic implants. Our study improves on the earlier studies by giving a more realistic scenario on ACB with knee prosthesis trials in place.

Our study has certain limitations. The adductor canal is a fascial plane and not a patent canal through which the neurovascular bundle passes. In such a closed space there is risk of entering the femoral vessels. The ultrasound-guided placement is the ideal way to ensure safety and avoid an intravascular injection; however, as previously described by anaesthesiologists, the standard aspiration and injection technique may be adopted during a DACB to confirm that there is no intravascular spread. Furthermore, the use of inflated tourniquets during TKA may overcrowd the tissues and obliterate the adductor canal. Also, how the injection will spread in patients cannot be stated by this current study. Hence, the real effect on pain improvement can only be ascertained by clinical studies. Clinical randomised studies may be needed to compare the efficacy between a USG-guided and a surgeon-administered ACB block.

## Conclusion

The radiographic analysis done as a part of this study described the landmarks of the adductor canal in relation to the knee joint and determined the trajectory of the surgeon-administered DACB. The cadaveric study demonstrated the feasibility and safety of a DACB as a part of the PAI during TKA.

## Data Availability

Not applicable
